# Greater municipal oral healthcare coverage is associated with lower odds of poor oral health among 5-year-old children in Brazil

**DOI:** 10.1590/1980-549720260002.supl.1

**Published:** 2026-06-26

**Authors:** Érica Torres de Almeida Piovesan, Luiza de Marilac Meireles Barbosa, Patrícia Maria Fonseca Escalda, Eduardo Bernabe

**Affiliations:** I Universidade de Brasília, Faculty of Health Sciences, Department of Dentistry - Brasilia (DF), Brazil.; II Universidade de Brasília, Faculty of Health Sciences and Technologies - Brasília (DF), Brazil.; III Queen Mary University of London, Institute of Dentistry - London, United Kingdom.

**Keywords:** Universal health coverage, Dental caries, Dental care for children, Urgent care, Child preschool, Brazil

## Abstract

**Objective:**

To investigate the association between municipal oral healthcare coverage and poor oral health among pre-school children.

**Methods:**

Individual-level data from 5-year-old children who participated in the National Oral Health Survey - SB Brasil 2023 were merged with municipal-level data on oral healthcare coverage, retrieved from the e-Gestor Atenção Primária à Saúde (e-Gestor APS). Poor oral health was determined by the presence of primary teeth with pulp involvement, ulceration, fistula and abscess (pufa prevalence) and the need for urgent dental care according to the World Health Organization (WHO) criteria. Four trajectories of municipal oral health coverage were identified using group-based trajectory modelling, namely always-low (reference group), medium-low, medium-high and always-high coverage. Multilevel logistic regression models were used to estimate associations adjusting for municipality and individual-level covariates.

**Results:**

The variance partition coefficient of the empty model (no predictors) indicated that 18.3 and 18.0% of the total individual variance in pufa prevalence and urgent dental care need, respectively, was due to differences between municipalities. Compared to children in municipalities with always-low coverage, those in municipalities with medium-high (odds ratio: 0.59, 95% confidence interval - 95%CI 0.35-0.98) and medium-low (0.63, 95%CI 0.40-0.99) coverage had lower odds of experiencing clinical consequences of untreated dental caries. Likewise, children in municipalities with always-high coverage (OR 0.49; 95%CI 0.27-0.89) had lower odds of needing urgent dental care than those in municipalities with always-low coverage.

**Conclusion:**

Greater municipal oral healthcare coverage was associated with lower odds of poor oral health among Brazilian pre-school children.

## INTRODUCTION

Global efforts to improve population health increasingly focus on achieving Universal Health Coverage (UHC), a strategy that seeks to increase access to healthcare while reducing financial barriers^1^ ^,^ ^2^. There is robust evidence that UHC positively impacts a wide range of health outcomes, from lower morbidity to reduced mortality^3^ ^,^ ^4^. Within this comprehensive approach, there is a growing consensus that oral healthcare services must be fully integrated to achieve truly equitable health^5^ ^,^ ^6^. Despite clear calls from the global health community, a significant challenge remains: a lack of empirical evidence directly linking UHC policies to population-level improvements in oral health outcomes^7^.

In Brazil, progress toward UHC has been achieved through the National Health System (Sistema Único de Saúde - SUS)^8^ The Family Health Strategy (Estratégia Saúde da Família - ESF) forms the foundation of the country’s primary care strategy and has progressively integrated oral healthcare services through the National Oral Health Policy (Brasil Sorridente)^9^ ^,^ ^10^ ^,^ ^11^ ^,^ ^12^. This policy expanded federal investment and incorporated oral health teams into family health teams, offering services from health promotion to diagnosis and treatment^11^ ^,^ ^13^. Despite a decreasing trend in dental caries rates compared to previous surveys, the National Oral Health Survey - SB Brasil 2023 indicates that dental caries among 5-year-olds remains a significant public health problem^14^.

Dental caries can lead to pain, infection, and negative impacts on quality of life, often requiring urgent dental treatment. The pufa index evaluates the clinical consequences of untreated dental caries by recording pulpal involvement (p), ulceration (u), fistula (f), and abscess (a)^15^ ^,^ ^16^. High pufa scores are more than just a clinical metric; they reflect delayed care and persistent health inequalities, making them a critical indicator for public health planning and policy evaluation. According to the National Oral Health Survey - SB Brasil 2023, the prevalence of children with clinical consequences of untreated dental caries (pufa≥1) was 13.98% among 5-year-olds^14^.

Evidence on the impact of oral healthcare coverage on pre-school children is limited. Determining whether increased coverage reduces the likelihood of experiencing clinical consequences of untreated dental caries and needing urgent dental care is vital for developing effective oral health policies and ensuring that expanding access results in meaningful reductions in the severe impacts of dental disease in early childhood. This study investigates the association between municipal oral healthcare coverage and poor oral health among Brazilian pre-school children. It was hypothesized that children in municipalities with higher oral healthcare coverage would have lower odds of experiencing clinical consequences of untreated dental caries and needing urgent dental care.

## METHODS

### Data sources

This cross-sectional study utilized two primary data sources: individual-level data from the National Oral Health Survey - SB Brasil 2023 and municipality-level data on oral healthcare coverage from multiple sources. The SB Brasil 2023 survey was conducted by the Brazilian Ministry of Health. It used a stratified, cluster random sampling approach to recruit a national sample of individuals from private permanent households in urban areas across the country. The target age groups were 5, 12, 15-19, 35-44, and 65-74 years. The survey protocol for SB Brasil 2023 was approved by the National Research Ethics Committee (reference 4.823.054). Informed consent was obtained from guardians, and informed assent was provided by the children themselves^17^. A total of 7,198 five-year-old children participated in the survey, with 7,185 undergoing clinical dental examinations.

### Measures

The primary outcome of this study was poor oral health, which was determined by primary care dentists using two clinical indicators. The first was the count of primary teeth with pulp involvement, ulceration of the oral mucosa due to carious lesions, fistula, or abscess (pufa index)^15^ ^,^ ^16^. Children with a pufa score greater than zero were considered as having clinical consequences of dental caries (pufa≥1). Examiners completed both theoretical and practical training sessions prior to the survey. The inter-examiner reliability was assessed and confirmed to be high, with weighted Kappa scores ranging from 0.61 to 1.00 for the pufa index^18^. Examiners also recorded whether the child needed immediate (urgent) treatment due to pain or infection of dental and/or oral origin, following the World Health Organization (WHO) criteria^19^.

**Figure 1.**
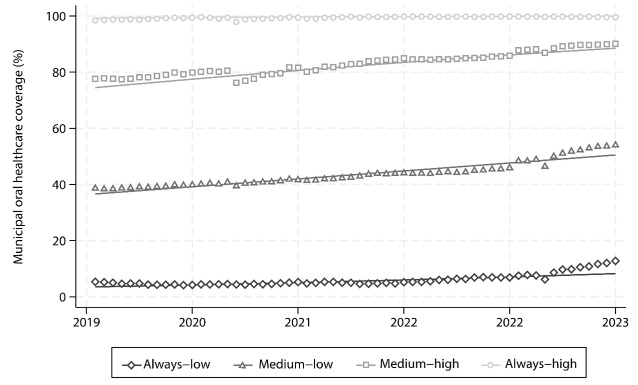
Trajectories of municipal oral healthcare coverage as part of the Family Health Strategy in Brazil, from January 2019 to December 2023 (n=5,570 municipalities). The four trajectories were identified using group-based trajectory modelling (see Methods).

The exposure was trajectories of municipal oral healthcare coverage from January 2019 to December 2023. Oral healthcare coverage was calculated by dividing the population registered with oral health teams by the total municipal population in a given month, according to government-established criteria^20^ ^,^ ^21^. Monthly data on oral healthcare coverage for all 5,570 municipalities in Brazil were retrieved from the e-Gestor Atenção Primária à Saúde (e-Gestor APS) database (https://egestoraps.saude.gov.br/)^22^ ^,^ ^23^. Four distinct trajectories of municipal oral healthcare coverage were empirically identified through group-based trajectory modelling (Figure 1).

These were

1. 2,640 municipalities (47.4%) which achieved full coverage in 2019 and sustained it through 2023 (always-high coverage);
2. 1,357 municipalities (24.4%) which started with high but incomplete coverage in 2019 and showed modest growth, nearly reaching full coverage by 2023 (medium-high coverage);
3. 1,019 municipalities (18.3%) which began with less than 50% coverage but gradually improved, surpassing the halfway mark (medium-low coverage); and
4. 554 municipalities (9.9%) which consistently maintained below 20% coverage throughout the study period (always-low coverage)^24^ .

The analysis included municipality- and individual-level factors as potential covariates. Municipal-level covariates included gross domestic product (GDP) per capita, geographical region (North, Northeast, Southeast [reference group], South, Central West), and municipality type (state capital [reference group] vs. interior). GDP per capita data for each municipality in 2019, expressed in increments of R$1,000, were obtained from the Brazilian Institute of Geography and Statistics (IBGE). Individual-level covariates included the child’s demographic characteristics (sex and ethnicity), and family socioeconomic status (maternal education and household income). Maternal education was recorded as years of completed full-time schooling and classified into three categories: basic (0-8 years), secondary (9-11 years), or higher education (12 or more years). Household income was expressed in terms of Brazilian minimum living wages (MLWs) and grouped as ≤1 MLW, >1 but ≤2 MLWs, and >2 MLWs.

### Statistical analysis

All analyses were conducted using Stata release 18.0 (StataCorp LLC, College Station, TX, USA), accounting for the survey’s complex design (stratification, clustering, and survey weights). To evaluate the impact of missing data, the characteristics of children with complete and missing data were compared using the standardized difference (SD). An SD value higher than 0.05 indicates differences between groups. Missing data on covariates were handled using multiple imputation by chained equations (MICE), assuming the data were missing at random^25^. The imputation model included all variables in the substantive analysis, auxiliary variables predictive of missingness (the sum of decayed, missing and filled primary teeth) and survey design features^26^ ^,^ ^27^. Fifty imputed datasets were generated, with imputations performed every 100 iterations. Each imputed dataset was analyzed separately, and results were combined using Rubin’s rules to produce estimates that accounted for imputation uncertainty^25^ ^,^ ^28^.

The associations of municipal oral healthcare coverage with pufa prevalence and urgent dental care need were assessed in separate multilevel logistic regression models. This approach was chosen because it is appropriate for binary outcomes and accounts for the hierarchical data structure, where children (level 1) are nested within municipalities (level 2). Odds ratios (ORs) were reported as the measure of association. Models were fitted sequentially to examine the contribution of different predictors. First, a random intercept model with no predictors (Model 0) was fitted to estimate the variance in the outcome at the municipality level. The variance partition coefficient (VPC) and median odds ratio (MOR) of this model were reported to quantify the municipality-level variance in each binary outcome^29^. VPC measures the total amount of variability in the outcome (on the log odds scale) attributed to differences between municipalities, while the MOR estimates the median increased in the odds of an outcome if an individual moves from a lower-risk to a higher-risk municipality. The higher the VPC and MOR values, the greater the variance at municipality level^29^. Next, Model 1 included all individual-level covariates, and Model 2 further incorporated all municipality-level predictors including municipal oral healthcare coverage. For each successive model, the proportional change in variance (PCV) was calculated to determine how much of the initial municipality-level variance was explained by the included predictors.

## Data availability statement:

Individual-level data from the National Oral Health Survey - SB Brasil 2023 are restricted due to privacy. Enquiries can be directed to the Ministry of Health of Brazil or the corresponding author. Monthly municipal oral healthcare coverage data are publicly available at https://egestoraps.saude.gov.br/.

## RESULTS

A total of 7,185 five-year-old children from 351 municipalities were included in the analyses. All children had complete data on outcomes, exposure and municipality-level covariates. However, 2,388 children (33.2%) had missing data on household income, 639 (8.9%) on maternal education and 147 (2.0%) on ethnicity. The extent and patterns of missing data are shown in Table S1 and Table S2, respectively. Overall, 4,520 (62.9%) had complete data on all relevant variables. There were differences between children with complete and missing data in terms of their ethnicity, maternal education and household income, but not in terms of oral health outcomes (Table 1). The prevalence of children with clinical consequences of untreated dental caries and urgent dental care need are shown according to municipal oral healthcare coverage in Table 2. There was no evidence of monotonic trends in oral health outcomes by trajectories.

**Table 1.** Comparison between children with complete (n=4,520) and missing data (n=2,650).

	Complete data		Missing data		Std. Diff.
	n*	%	n*	%	
Sex					
Male	2,313	49.0	1,357	48.6	0.01
Female	2,207	51.0	1,308	51.5	-0.01
Race/ethnicity					
White	1,569	44.7	919	42.9	0.04
Black	419	9.3	319	12.7	-0.11
Mixed	2,475	44.6	1,237	43.6	0.02
Asian	38	0.8	38	0.9	-0.01
Indigenous	19	0.6	5	0.0	0.11
Mother’s education level					
Basic	1,556	35.1	726	42.1	-0.14
Secondary	2,190	44.7	959	43.8	0.02
Higher	774	20.2	341	14.1	0.16
Household income					
≤1 MLW	1,568	31.8	118	40.6	-0.19
>1 but ≤2 MLWs	1,499	31.8	79	27.7	0.09
>2 MLWs	1,453	36.4	80	31.6	0.10
Pufa prevalence					
Pufa score=0	3,891	86.1	2,318	85.9	0.01
Pufa score≥1	629	13.9	347	14.2	-0.01
Urgent dental care					
No needed	3,993	89.0	2,400	89.2	-0.01
Needed	527	11.0	265	10.8	0.01

Std Diff: standardised difference; MLW: minimum living wage; Pufa: pulp involvement, ulceration, fistula or abscess.

*Counts are unweighted.

**Table 2.** Prevalence of children with clinical consequences of untreated dental caries (pufa≥1) and urgent dental care need according to trajectories of municipal oral healthcare coverage.

	Pufa prevalence		Urgent dental care need	
	%	(95%CI)	%	(95%CI)
Always-low	14.20	(10.20-18.20)	13.32	(9.33-17.30)
Medium-low	11.72	(9.16-14.27)	9.74	(7.58-11.90)
Medium-high	16.55	(13.10-20.00)	12.49	(9.31-15.66)
Always-high	19.08	(13.43-24.74)	10.31	(6.61-14.00)
p-value for trend*		0.524		0.535

*The p-value for the linear trend was derived from a crude two-level logistic regression model treating coverage trajectories as a continuous predictor.

The multilevel models for the association between municipal oral healthcare coverage and pufa prevalence are presented in Table 3. The VPC of the empty model indicated that 18.3% of the variance in the outcome was attributable to differences between municipalities. Including all individual-level covariates in Model 1 reduced the municipality-level variance by 4.8%, while the subsequent addition of municipality-level predictors, including municipal oral healthcare coverage, explained 14.7% of the remaining variance. Children in municipalities with medium-high (0.59; 95%CI 0.35-0.98) and medium-low (0.63; 95%CI 0.40-0.99) coverage had lower odds of experiencing clinical consequences of untreated dental caries than those in municipalities with always-low coverage. However, no difference was found between children in municipalities with always-high and always-low coverage (Table 3).

**Table 3.** The association between municipal oral healthcare coverage and the prevalence of 5-year-old children with clinical consequences of dental caries (pufa≥1) (n=7,185 children in 351 municipalities).

Fixed effects	Model 0	Model 1	Model 2
	OR (95%CI)	OR (95%CI)	OR (95%CI)
Municipal oral healthcare coverage			
Always-low			1.00 (Reference)
Medium-low			0.63 (0.40-0.99)
Medium-high			0.59 (0.35-0.98)
Always-high			0.63 (0.33-1.20)
Municipal GDP per capita, per R$1000-increase			0.99 (0.98-1.00)
Geographical region			
North			2.22 (1.31-3.78)
Northeast			1.32 (0.77-2.30)
Southeast			1.00 (reference)
South			1.56 (0.86-2.83)
Central West			1.54 (0.89-2.66)
Municipality type			
State capital			1.00 (reference)
Non-capital			1.27 (0.89-2.66)
Random effects	Estimates	Estimates	Estimates
Variance (SE)	0.74 (0.15)	0.70 (0.16)	0.60 (0.17)
MOR	2.27	2.22	2.09
VPC-level 2	18.3%	17.6%	15.4%
PCV		-4.8%	-14.7%

CI: confidence interval; GDP: municipal-level gross domestic product; MOR: median odds ratio; PCV: percentual change in variance: Pufa: pulp involvement, ulceration, fistula and abscess; OR: odds ratio; SE: standard error; VPC-level 2: variance partition coefficient at municipality level.

A two-level logistic regression model was fitted with children nested within municipalities. Model 0 was empty (no predictors). Model 1 included all individual-level covariates as predictors. Model 2 included all individual- and municipality-level covariates and municipal oral healthcare coverage as predictors.

Results for urgent dental care need are shown in Table 4. The VPC of the empty model indicated that 18.0% of the outcome variance was attributable to differences between municipalities, which decreased by 4.0% after inclusion of individual-level covariates in Model 1. The subsequent inclusion of municipality-level predictors, including municipal oral healthcare coverage, in the model accounted for 6.9% of the remaining variance (Model 2). Children in municipalities with always-high coverage (OR 0.50; 95%CI 0.25-0.99) had lower odds of needing urgent dental care than those in municipalities with always-low coverage. However, there were no differences between children in municipalities with medium-high and medium-low coverage and those in municipalities with always-low coverage (Table 4).

**Table 4.** The association between municipal oral healthcare coverage and prevalence of 5-year-old children with urgent dental care need (n=7,185 children in 351 municipalities).

Fixed effects	Model 0	Model 1	Model 2
	OR (95%CI)	OR (95%CI)	OR (95%CI)
Municipal oral healthcare coverage			
Always-low			1.00 (Reference)
Medium-low			0.69 (0.41-1.14)
Medium-high			0.76 (0.43-1.36)
Always-high			0.50 (0.25-0.99)
Municipal GDP per capita, R$1000-increase			1.00 (0.99-1.01)
Geographical region			
North			1.63 (0.90-2.96)
Northeast			1.06 (0.58-1.91)
Southeast			1.00 (Reference)
South			1.29 (0.66-2.54)
Central West			1.44 (0.77-2.71)
Municipality type			
State capital			1.00 (Reference)
Interior			1.05 (0.72-1.53)
Random effects	Estimates	Estimates	Estimates
Variance (SE)	0.72 (0.22)	0.70 (0.25)	0.65 (0.24)
MOR	2.25	2.22	2.15
VPC-level 2	18.0%	17.4%	16.4%
PCV		-4.0%	-6.9%

CI: confidence interval; GDP: municipal-level gross domestic product; MOR: median odds ratio; PCV: percentual change in variance; OR: odds ratio; SE: standard error; VPC-level 2: variance partition coefficient at municipality level.

A two-level logistic regression model was fitted with children nested within municipalities. Model 0 was empty (no predictors). Model 1 included all individual-level covariates as predictors. Model 2 included all individual- and municipality-level covariates and municipal oral healthcare coverage as predictors.

## DISCUSSION

The findings of this study indicated that, compared to children living in municipalities with always-low oral healthcare coverage, those in municipalities with medium-low and medium-high coverage were less likely to experience clinical consequences of untreated dental caries, whereas those in municipalities with always-high coverage were less likely to require urgent dental care, after accounting for covariates at both the municipal and individual levels.

Crude geographical variation at the municipal level explained 18.3% of the total individual variance in pufa prevalence and 18.0% in urgent need for dental care. After accounting for the sociodemographic composition of municipalities (individual-level predictors), these VPCs decreased slightly to 17.6 and 17.4%, respectively. Since VPCs≥5% are typically interpreted as reflecting moderate contextual effects^30^, these findings suggest that the municipal context plays a meaningful role in shaping oral health outcomes among young children. The considerable oral health inequalities observed between municipalities may reflect variations in long-term investment in infrastructure, public health programs, or access to care. The inclusion of municipality-level predictors, including municipal oral healthcare coverage, accounted for some but not all the total individual level variance in pufa prevalence (PCV=14.7%) and urgent dental care need (PCV=6.9%), suggesting that additional contextual factors, such as the structural determinants of health, may also contribute to these inequalities.

Social, political and economic systems can influence children’s oral health through policies, resource allocation and the organization of society. Social policies, including the availability and quality of public schooling (especially during nursery and preschool years), and welfare systems (e.g. government spending on health and social protection) create institutional opportunities for oral health promotion, prevention and early management of dental diseases. They are therefore critical to facilitate timely treatment and prevent progression to acute dental emergencies.

The present findings demonstrate that expanding municipal oral healthcare coverage can reduce, but does not completely eliminate, geographical inequalities in oral health. Simply having services available does not guarantee effective access, as service priorities and workforce distribution can affect the scope and quality of care^31^. The variability in oral health outcomes seen across municipalities highlights the need to consider not only service availability but also their effectiveness in delivering age-appropriate preventive and restorative care. Another challenge is the predominant model of care, where oral health professionals mainly operate within dental offices, with limited collaboration with the education sector to provide sustained oral health promotion and oral disease prevention. Government initiatives, such as the Programa Saúde na Escola, which aims to integrate primary care with educational settings, have struggled to achieve consistent and broad implementation. Addressing these gaps is crucial to maximizing the preventive impact of municipal coverage^11^ ^,^ ^12^.

The expected protective role of expanded oral healthcare coverage within SUS has been widely recognised. The ESF and the National Oral Health Policy (Brasil Sorridente) were designed to reduce inequalities in access to dental care, and recent studies have documented the expansion and consolidation of oral health teams within primary care^32^ ^,^ ^33^. These teams provide a wide range of free services, including health promotion, oral disease prevention, diagnosis, restorative care, extractions, and emergency care^13^. Previous studies have shown that children in areas served by ESF health centres are more likely to visit the dentist early in life, report better oral health-related quality of life and receive more dental treatment (fillings and extractions) than their counterparts^34^ ^,^ ^35^. The present findings are significant because the analysis period (2019-2023) encompassed the COVID-19 pandemic, which disrupted Brazil’s healthcare system from 2020 to 2022. During that time, elective procedures were suspended, and oral health teams were reassigned to urgent care. Despite these challenges, urgent dental care continued to be provided through the pandemic.

These findings have some implications for policy. They support the notion that expanding municipal oral healthcare coverage can help reduce the prevalence of children with poor oral health. In a system grounded in universal health coverage, achieving broad and equitable municipal coverage is fundamental to uphold the principles of primary health care. Strengthening and maintaining oral healthcare coverage should therefore remain a priority to ensure timely access to care and to promote more equitable oral health outcomes for young children in Brazil.

This study has some limitations. First, despite having longitudinal data on municipal oral healthcare coverage, the cross-sectional nature of the SB Brasil survey precludes us from making any causal inferences regarding the impact of ESF on childhood oral health. Second, the analysis assumed that children had resided in the same municipality since they were born. Differential misclassification of exposure could have occurred if patterns of residential mobility varied by oral health outcomes. In addition, residual confounding cannot be excluded, especially due to unmeasured contextual factors such as availability of social programs and community water fluoridation.

The present study indicated that, compared to children living in municipalities with consistently high oral healthcare coverage, those in municipalities with medium coverage were less likely to experience clinical consequences of untreated dental caries, while those in municipalities with consistently high coverage were less likely to require urgent dental care. The findings also indicate that children in municipalities with consistently low coverage face greater odds of severe, yet preventable, dental disease. In a health system founded on the principles of universal health coverage, the failure to adequately serve young children constitutes a critical fault that warrants urgent attention.

## Supplementary Material

Supplementary Material

## References

[1] Moreno-Serra R, Smith PC (2012). Does progress towards universal health coverage improve population health?. Lancet.

[2] Ranabhat CL, Atkinson J, Park MB, Kim CB, Jakovljevic M (2018). The Influence of Universal Health Coverage on Life Expectancy at Birth and Healthy Life Expectancy: a multi-country cross-sectional study. Front Pharmacol.

[3] Kruk ME, Gage AD, Joseph NT, Danaei G, García-Saisó S, Salomon JA (2018). Mortality due to low-quality health systems in the universal health coverage era: a systematic analysis of amenable deaths in 137 countries. Lancet.

[4] Endalamaw A, Mengistu ST, Khatri RB, Wolka E, Erku D, Zewdie A (2025). Universal health coverage: exploring the what, how, and why using realist review. PLOS Glob Public Health.

[5] Wang TT, Mathur MR, Schmidt H (2020). Universal health coverage, oral health, equity and personal responsibility. Bull World Health Organ.

[6] Watt RG, Daly B, Allison P, Macpherson LMD, Venturelli R, Listl S (2019). Ending the neglect of global oral health: time for radical action. Lancet.

[7] Santos SQM, Andrade RVS, Galvão MHR, Oliveira AGRC (2024). Oral health approach in universal health coverage. BMC Public Health.

[8] Castro MC, Massuda A, Almeida G, Menezes-Filho NA, Andrade MV, Noronha KVMS (2019). Brazil’s unified health system: the first 30 years and prospects for the future. Lancet.

[9] Bastos ML, Menzies D, Hone T, Dehghani K, Trajman A (2017). The impact of the Brazilian family health strategy on selected primary care sensitive conditions: a systematic review. PLoS One.

[10] D’Avila OP, Chisini LA, Costa FS, Cademartori MG, Cleff LB, Castilhos ED (2021). Use of health services and family health strategy households population coverage in Brazil. Ciênc Saúde Coletiva.

[11] Pucca GA, Gabriel M, Araujo ME, Almeida FCS (2015). Ten years of a National Oral Health Policy in Brazil: innovation, boldness, and numerous challenges. J Dent Res.

[12] Cayetano MH, Carrer FCA, Gabriel M, Martins FC, Pucca GA, Araujo ME (2019). Política Nacional de Saúde Bucal Brasileira (Brasil Sorridente): um resgate da história, aprendizados e futuro. Univ Odontol.

[13] Trezena S, Oliveira FES, Dias VO, Martelli PJL, Martelli DRB, Martelli H (2023). Specialized dental care in the Brazilian Unified National Health System (SUS). Pesqui Bras Odontopediatria Clín Integr.

[14] Brasil. Ministério da Saúde. Secretaria de Atenção Primária à Saúde. Departamento de Estratégias e Políticas de Saúde Comunitária (2025). SB Brasil 2023: Pesquisa Nacional de Saúde Bucal: relatório final.

[15] Monse B, Heinrich-Weltzien R, Benzian H, Holmgren C, van Palenstein Helderman W (2010). PUFA--an index of clinical consequences of untreated dental caries. Community Dent Oral Epidemiol.

[16] Monse B, Heinrich-Weltzien R, Benzian H, Holmgren C, van Palenstein Helderman W (2010). The PUFA index: an index for measuring untreated dental caries in children. Community Dent Oral Epidemiol.

[17] Alves MCGP, Alencar GP, Vargas AMD, Ferreira RC, Bernal RTI (2025). Sampling plan of SB Brasil 2023: precision of dmft and DMFT estimates for the study domains. Braz Oral Res.

[18] Ferreira RC, Pinto RS, Reis C, Moura RNV, Aguiar SO, Drummond AMA (2025). Calibration of SB Brasil 2023 examiners: use of technologies associated with the In-Lux method. Braz Oral Res.

[19] World Health Organization (2013). Oral health surveys: basic methods.

[20] Nagin DS, Odgers CL (2010). Group-based trajectory modeling in clinical research. Annu Rev Clin Psychol.

[21] Sinha P, Calfee CS, Delucchi KL (2021). Practitioner’s guide to latent class analysis: methodological considerations and common pitfalls. Crit Care Med.

[22] Brasil. Ministério da Saúde. Secretaria de Ateção à Saúde. Departamento de Atenção Básica (2025). Novo método de cálculo do indicador. Cobertura populacional estimada pela Saúde Bucal na Atenção Básica.

[23] Silva AS, Laprega MR (2005). Critical evaluation of the Primary Care Information System (SIAB) and its implementation in Ribeirao Preto, Sao Paulo, Brazil. Cad Saude Publica.

[24] Piovesan ETA, Bernabe E (2025). Municipal oral healthcare coverage and dental caries among Brazilian children. Caries Res.

[25] White IR, Royston P, Wood AM (2011). Multiple imputation using chained equations: Issues and guidance for practice. Stat Med.

[26] Quartagno M, Carpenter JR, Goldstein H (2019). Multiple Imputation with survey weights: a multilevel approach. J Surv Stat Methodol.

[27] De Silva AP, De Livera AM, Lee KJ, Moreno-Betancur M, Simpson JA (2021). Multiple imputation methods for handling missing values in longitudinal studies with sampling weights: comparison of methods implemented in Stata. Biom J.

[28] Austin PC, White IR, Lee DS, van Buuren S (2021). Missing data in clinical research: a tutorial on multiple imputation. Can J Cardiol.

[29] Merlo J, Chaix B, Ohlsson H, Beckman A, Johnell K, Hjerpe P (2006). A brief conceptual tutorial of multilevel analysis in social epidemiology: using measures of clustering in multilevel logistic regression to investigate contextual phenomena. J Epidemiol Community Health.

[30] Merlo J, Wagner P, Leckie G (2019). A simple multilevel approach for analysing geographical inequalities in public health reports: the case of municipality differences in obesity. Health Place.

[31] Ribeiro AGA, Martins RFM, Vissoci JRN, Silva NC, Rocha TAH, Queiroz RCS (2021). Progress and challenges in potential access to oral health primary care services in Brazil: a population-based panel study with latent transition analysis. PLoS One.

[32] Menezes LXB, Corrêa GT, Lucena EHG, Celeste RK, Cavalcanti YW (2025). Time trend of Family Health Strategy oral health teams in Brazilian municipalities from 2001 to 2021. Cad Saude Publica.

[33] Lucena EHG, Lucena CDRX, Alemán JAS, Pucca GA, Pereira AC, Cavalcanti YW (2020). Monitoring of oral health teams after National Primary Care Policy 2017. Rev Saude Publica.

[34] Feldens CA, Fortuna MJ, Kramer PF, Ardenghi TM, Vítolo MR, Chaffee BW (2018). Family Health Strategy associated with increased dental visitation among preschool children in Brazil. Int J Paediatr Dent.

[35] Moraes RB, Sfreddo CS, Ardenghi TM (2021). Impact of the Brazilian Family Health Strategy on child oral health-related quality of life: a cohort study. Braz Oral Res.

