# Supplementary material for: Greater municipal oral healthcare coverage is associated with lower odds of poor oral health among 5-year-old children in Brazil
Source: Rev Bras Epidemiol. 2026 Jun 26;29(Suppl 1):e260002supl1. doi: 10.1590/1980-549720260002.supl.1 (PMC13317029; doi:10.1590/1980-549720260002.supl.1)
Supplement: Supplementary file 1 [file 1980-5497-rbepid-29-suppl1-e260002supl1-sppl.pdf]

Table S1. Extent of missing data

Number of variables with missing data	n	%	Cumulative %
None	4,520	62.9	62.9
1 variable	2,209	30.7	93.7
2 variables	403	5.6	99.3
3 variables	53	0.7	100.0
Total	7,185	100.0	

Table S2. Patterns of missing data

# variables with complete data	Variables			Number of participants
	Ethnicity	Maternal education	Household income	
3	1	1	1	4,520
2	1	1	0	1,942
2	1	0	1	226
2	0	1	1	41
1	1	0	0	350
1	0	1	0	43
1	0	0	1	10
0	0	0	0	53

0 means data is missing; 1 means data is present.
