# Supplementary material for: Greater municipal oral healthcare coverage is associated with lower odds of poor oral health among 5-year-old children in Brazil
Source: Rev Bras Epidemiol. 2026 Jun 26;29(Suppl 1):e260002supl1. doi: 10.1590/1980-549720260002.supl.1 (PMC13317029; doi:10.1590/1980-549720260002.supl.1)
Supplement: Translation

# Translation: Maior cobertura municipal de saúde bucal está associada a menores chances de má saúde bucal em crianças de cinco anos no Brasil

**DOI:** 10.1590/1980-549720260002.supl.1.2

**Authors:** Érica Torres de Almeida Piovesan, Luiza de Marilac Meireles Barbosa, Patrícia Maria Fonseca Escalda, Eduardo Bernabe

**Affiliations:** I Universidade de Brasília, Faculdade de Ciências da Saúde, Departamento de Odontologia - Brasília (DF), Brasil.; II Universidade de Brasília, Faculdade de Ciências e Tecnologias em Saúde - Brasília (DF), Brasil.; III Queen Mary University of London, Institute of Dentistry - Londres, Reino Unido.

**Keywords:** Cobertura universal de saúde, Cárie dentária, Pré-escolares, Tratamento de urgência, Odontopediatria, Brasil

## Abstract

**Objetivo:**

Investigar a associação entre a cobertura municipal de saúde bucal e a má saúde bucal entre pré-escolares.

**Métodos:**

Dados em nível individual de crianças de cinco anos do SB Brasil 2023 foram combinados com dados municipais de cobertura de saúde bucal, obtidos do e-Gestor APS. A má saúde bucal foi medida pelo índice pufa e pela necessidade de tratamento odontológico de urgência, de acordo com os critérios da Organização Mundial da Saúde (OMS). Quatro trajetórias de cobertura municipal foram identificadas usando modelagem de trajetória baseada em grupos: cobertura sempre-baixa (referência), média-baixa, média-alta e sempre-alta. Modelos de regressão logística multinível foram usados para estimar associações, ajustando para covariáveis em nível municipal e individual.

**Resultados:**

O coeficiente de partição da variância do modelo nulo (sem preditores) demonstrou que 18,3 e 18,0% da variância individual em pufa e a necessidade de urgência odontológica, respectivamente, foram devidos a diferenças entre os municípios. Em comparação com crianças em municípios com cobertura sempre-baixa, aquelas em áreas de cobertura média-alta (*odds ratio -* OR 0,59, intervalo de confiança de 95% - IC95% 0,35-0,98) e média-baixa (OR 0,63, IC95% 0,40-0,99) tiveram menor chance de apresentar consequências clínicas de cárie dentária não tratada (pufa≥1). Da mesma forma, crianças em municípios de cobertura sempre-alta (OR 0,49; IC95% 0,27-0,89) tiveram menor chance de necessidade de urgência odontológica do que aquelas em áreas de cobertura sempre-baixa.

**Conclusão:**

Maior cobertura municipal de saúde bucal esteve associada a menores chances de má saúde bucal entre pré-escolares brasileiros.

## INTRODUÇÃO

Os esforços globais para melhorar a saúde da população se concentram cada vez mais em alcançar a cobertura universal de saúde (do inglês, *Universal Health Coverage* - UHC), uma estratégia que busca aumentar o acesso aos serviços de saúde, reduzindo as barreiras financeiras^1^ ^,^ ^2^. Há evidências sólidas de que a UHC impacta positivamente uma ampla gama de desfechos em saúde, desde menor morbidade até redução de mortalidade^3^ ^,^ ^4^. Dentro desta abordagem abrangente, há um consenso crescente de que os serviços de saúde bucal devem ser totalmente integrados para alcançar uma saúde verdadeiramente equitativa^5^ ^,^ ^6^. Apesar dos apelos claros da comunidade global de saúde, um desafio relevante permanece: a falta de evidências empíricas que vinculam diretamente as políticas de UHC a melhorias em nível populacional nos desfechos de saúde bucal^7^.

No Brasil, o progresso em direção à UHC foi alcançado através do Sistema Único de Saúde (SUS)^8^. A Estratégia Saúde da Família (ESF) constitui a base da estratégia de Atenção Básica do país e tem integrado progressivamente os serviços de saúde bucal por meio da Política Nacional de Saúde Bucal (Brasil Sorridente)^9^ ^,^ ^10^ ^,^ ^11^ ^,^ ^12^. Essa política ampliou o investimento federal e incorporou as equipes de saúde bucal nas equipes de saúde da família, oferecendo serviços desde a promoção de saúde até o diagnóstico e o tratamento^11^ ^,^ ^13^. Apesar de uma tendência decrescente das taxas de cárie dentária em comparação com inquéritos anteriores, de acordo com a Pesquisa Nacional de Saúde Bucal - SB Brasil 2023, a cárie dentária entre crianças de cinco anos continua a ser um grave problema de saúde pública^14^.

Lesões de cárie podem resultar em dor, infecção e impactos negativos na qualidade de vida, muitas vezes exigindo tratamento odontológico de urgência. O índice pufa avalia as consequências clínicas de cárie dentária não tratada por meio do registro do envolvimento pulpar (p), ulceração (u), fístula (f), e abscesso (a)^15^ ^,^ ^16^. Os altos valores do índice pufa são mais do que apenas uma métrica clínica; eles refletem o atraso na assistência e as desigualdades persistentes na saúde, tornando-se um indicador crítico para o planejamento da saúde pública e a avaliação de políticas. De acordo com a Pesquisa Nacional de Saúde Bucal - SB Brasil 2023, a prevalência de crianças com consequências clínicas de cárie dentária não tratada (pufa≥1) foi de 13,98% entre as crianças de cinco anos^14^.

Evidências sobre o impacto da cobertura de saúde bucal em pré-escolares são limitadas. Determinar se o aumento da cobertura reduz a probabilidade de sofrer consequências clínicas de cárie dentária não tratada e a necessidade de tratamento odontológico de urgência é fundamental para o desenvolvimento de políticas de saúde bucal eficazes e para garantir que a expansão do acesso resulte em reduções significativas nas repercussões graves da doença dentária na primeira infância. Neste estudo, nós investigamos a associação entre a cobertura municipal de saúde bucal e má saúde bucal entre crianças brasileiras pré-escolares. Nossa hipótese é de que crianças dos municípios com maior cobertura de saúde bucal teriam menores chances de sofrer consequências clínicas de cárie dentária não tratada e necessitarem de tratamento odontológico de urgência.

## MÉTODOS

### Fontes de dados

Duas fontes de dados primárias foram utilizadas neste estudo transversal: dados em nível individual da Pesquisa Nacional de Saúde Bucal - SB Brasil 2023 e dados municipais de cobertura de saúde bucal de diversas fontes. A pesquisa SB Brasil 2023 foi realizada pelo Ministério da Saúde. Utilizou-se uma abordagem estratificada, por conglomerado, de amostragem aleatória para coletar uma amostra nacional de indivíduos com domicílios particulares permanentes em áreas urbanas de todo o país. As faixas etárias-alvo foram 5, 12, 15-19, 35-44 e 65-74 anos. O protocolo de pesquisa para o SB Brasil 2023 foi aprovado pela Comissão Nacional de Ética em Pesquisa (referência 4.823.054). O Consentimento Livre e Esclarecido foi obtido dos responsáveis, e o Assentimento Livre e Esclarecido foi obtido das crianças^17^. 7.198 crianças com cinco anos de idade participaram da pesquisa, dentre as quais 7.185 passaram por exames clínicos odontológicos.

### Medidas

A má saúde bucal foi o desfecho primário deste estudo, sendo determinada pelos dentistas da Atenção Básica usando dois indicadores clínicos. O primeiro foi a contagem de dentes decíduos com envolvimento pulpar, ulceração da mucosa oral devido a lesões cariosas, fístula ou abscesso (índice pufa)^15^ ^,^ ^16^. Crianças com um escore de pufa maior que zero foram consideradas como tendo consequências clínicas de cárie dentária (pufa≥1). Os avaliadores passaram por treinamento teórico e prático antes da pesquisa. A confiabilidade inter-avaliadores foi avaliada e confirmada como alta, com escore de Kappa ponderado variando de 0,61 a 1,00 para o índice pufa^18^. Os avaliadores também registraram se a criança precisava de tratamento imediato (urgente) por dor ou infecção de origem dentária e/ou bucal, seguindo os critérios da Organização Mundial de Saúde (OMS)^19^.

As trajetórias da cobertura municipal de saúde bucal no período de janeiro de 2019 a dezembro de 2023 consistiram na exposição. A cobertura de saúde bucal foi calculada dividindo-se a população registrada nas equipes de saúde bucal pela população municipal total em um determinado mês, segundo critérios estabelecidos pelo governo^20^ ^,^ ^21^. Os dados mensais sobre a cobertura de saúde bucal dos 5.570 municípios brasileiros foram obtidos da base de dados e-Gestor Atenção Primária à Saúde (e-Gestor APS) (https://egestoraps.saude.gov.br/)^22^ ^,^ ^23^. Quatro trajetórias distintas de cobertura municipal de saúde bucal foram empiricamente identificadas usando modelagem de trajetória baseada em grupos (Figura 1).

As trajetórias foram as seguintes:

1. 2.640 municípios (47,4%) que atingiram cobertura total em 2019 e a sustentaram até 2023 (cobertura sempre-alta);
2. 1.357 municípios (24,4%) que começaram com cobertura alta, porém incompleta em 2019 e apresentaram crescimento modesto, quase atingindo a cobertura total em 2023 (cobertura média-alta);
3. 1.019 municípios (18,3%) que começaram com menos de 50% de cobertura, mas que gradualmente melhoraram, ultrapassando a marca de 50% (cobertura média-baixa); e
4. 554 municípios (9,9%) que se mantiveram consistentemente abaixo dos 20% de cobertura ao longo do período estudado (cobertura sempre-baixa)^24^.

**Figura 1.** Trajetórias da cobertura municipal de saúde bucal como parte da Estratégia Saúde da Família no Brasil, de janeiro de 2019 a dezembro de 2023 (n=5.570 municípios). As quatro trajetórias foram identificadas usando modelagem de trajetória baseada em grupos (ver Métodos).

A análise incluiu fatores em níveis municipal e individual como covariáveis potenciais. As covariáveis em nível municipal incluíram o Produto Interno Bruto (PIB) *per capita*, região geográfica (Norte, Nordeste, Sudeste [grupo de referência], Sul, Centro-Oeste) e tipo de município (capital do estado [grupo de referência] *vs.* interior). Os dados do PIB *per capita* de cada município em 2019, expressos em acréscimos de R$ 1.000, foram obtidos do Instituto Brasileiro de Geografia e Estatística (IBGE). As covariáveis em nível individual incluíram as características demográficas da criança (sexo e etnia) e o nível socioeconômico familiar (escolaridade materna e renda familiar). A escolaridade materna foi registrada como anos completos de educação em tempo integral e classificada em três categorias: educação primária (0-8 anos), educação secundária (9-11 anos) ou educação superior (12 ou mais anos). A renda familiar foi expressa considerando o salário mínimo (SM) dos brasileiros e agrupado em ≤1 SM, >1 mas ≤2 SMs, e >2 SMs.

### Análise estatística

Todas as análises foram realizadas utilizando o software Stata versão 18.0 (StataCorp LLC, College Station, TX, EUA), abrangendo o desenho complexo da pesquisa (estratificação, agrupamento e pesos de pesquisa). Para avaliar o impacto de dados faltantes, as características de crianças com dados completos e dados faltantes foram comparadas por meio da Diferença Padronizada (DP). Um valor de DP superior a 0,05 indica diferenças entre grupos. Dados faltantes de covariáveis foram tratados usando a Imputação Múltipla por Equações Encadeadas (*Multiple Imputation by Chained Equations* - MICE), assumindo que os dados estavam ausentes de forma aleatória^25^. O modelo de imputação incluiu todas as variáveis na análise substantiva, variáveis auxiliares preditivas de carência (a soma de dentes decíduos cariados, perdidos e obturados/restaurados) e características do tipo de estudo^26^ ^,^ ^27^. Um total de 50 conjuntos de dados imputados foram gerados, considerando que as imputações foram realizadas a cada 100 iterações. Cada conjunto de dados imputados foi analisado separadamente, e os resultados foram combinados usando as regras de Rubin para produzir estimativas que representem a incerteza de imputação^25^ ^,^ ^28^.

As associações da cobertura municipal de saúde bucal com o índice pufa e a necessidade de tratamento odontológico de urgência foram avaliadas em modelos separados de regressão logística multinível. Esta abordagem foi escolhida por ser adequada para resultados binários e representar a estrutura hierárquica de dados, na qual as crianças (nível 1) estão aninhadas nos municípios (nível 2). As razões de chances (*odds ratio* - OR) foram apresentadas como medida de associação. Os modelos foram ajustados sequencialmente para avaliar a contribuição de diferentes preditores. Primeiro, um modelo nulo de intercepto aleatório (Modelo 0) foi ajustado para estimar a variância do desfecho em nível municipal. O coeficiente de partição da variância (CPV) e a razão de chance mediana (*Median Odds Ratio* - MOR) desse modelo foram apresentados para quantificar a variância em nível municipal em cada desfecho binário^29^. O CPV mede a quantidade total de variabilidade no desfecho (na escala de probabilidade logarítmica) atribuída às diferenças entre os municípios, enquanto a MOR estima que a mediana aumentou nas probabilidades de um desfecho caso um indivíduo mude de um município de menor risco para um município de maior risco. Quanto maiores os valores de CPV e MOR, maior a variância em nível municipal^29^. Em seguida, o Modelo 1 incluiu todas as covariáveis em nível individual, e o Modelo 2 incorporou todos os preditores em nível municipal, incluindo a cobertura municipal de saúde bucal. Para cada modelo sucessivo, a alteração proporcional na variância (APV) foi calculada para determinar quanto da variância inicial em nível municipal foi explicada pelos preditores incluídos.

## Declaração de disponibilidade de dados:

Os dados em nível individual da Pesquisa Nacional de Saúde Bucal - SB Brasil 2023 são restritos devido à privacidade. As consultas podem ser dirigidas ao Ministério da Saúde ou ao autor correspondente. Os dados mensais de cobertura municipal de saúde bucal estão disponíveis publicamente em https://egestoraps.saude.gov.br/.

## RESULTADOS

Um total de 7.185 crianças de cinco anos de idade, provenientes de 351 municípios, foram incluídas nas análises. Todas as crianças tinham dados completos sobre os desfechos, exposição e covariáveis em nível municipal. No entanto, 2.388 crianças (33,2%) tinham dados faltantes sobre a renda familiar, 639 (8,9%) sobre a escolaridade materna e 147 (2,0%) sobre a etnia. A extensão e os padrões dos dados faltantes são apresentados nas Tabela S1 e Tabela S2, respectivamente. Em geral, 4.520 (62,9%) tinham dados completos sobre todas as variáveis relevantes. Houve diferenças entre as crianças com dados completos e dados faltantes com relação à etnia, escolaridade materna e renda familiar, mas não com relação a desfechos de saúde bucal (Tabela 1). A prevalência de crianças com consequências clínicas de cárie dentária não tratada e necessidade de tratamento odontológico de urgência é apresentada na Tabela 2, segundo a cobertura municipal de saúde bucal. Não houve evidência de tendências monotônicas nos desfechos de saúde bucal por trajetórias.

**Tabela 1.** Comparação entre crianças com dados completos (n=4.520) e dados faltantes (n=2.650).

	Dados completos		Dados faltantes		Dif. Pad.
	n*	%	n*	%	
Sexo					
Masculino	2.313	49,0	1.357	48,6	0,01
Feminino	2.207	51,0	1.308	51,5	-0,01
Raça/etnia					
Branca	1.569	44,7	919	42,9	0,04
Negra	419	9,3	319	12,7	-0,11
Parda	2.475	44,6	1.237	43,6	0,02
Amarela	38	0,8	38	0,9	-0,01
Indígena	19	0,6	5	0,0	0,11
Escolaridade materna					
Educação Primária	1.556	35,1	726	42,1	-0,14
Educação Secundária	2.190	44,7	959	43,8	0,02
Educação Superior	774	20,2	341	14,1	0,16
Renda familiar					
≤1 SM	1.568	31,8	118	40,6	-0,19
>1 mas ≤2 SMs	1.499	31,8	79	27,7	0,09
>2 SMs	1.453	36,4	80	31,6	0,10
Índice pufa					
Escore pufa=0	3.891	86,1	2.318	85,9	0,01
Escore pufa≥1	629	13,9	347	14,2	-0,01
Tratamento odontológico de urgência					
Não necessário	3.993	89,0	2.400	89,2	-0,01
Necessário	527	11,0	265	10,8	0,01

Dif. Pad.: Diferença padronizada; SM: Salário mínimo; Pufa: Envolvimento pulpar, ulceração, fístula ou abscesso.

*Somas não ponderadas.

**Tabela 2.** Prevalência de crianças com consequências clínicas de cárie dentária não tratada (pufa≥1) e necessidade de tratamento odontológico de urgência de acordo com as trajetórias de cobertura municipal de saúde bucal.

	Índice pufa		Necessidade de tratamento odontológico de urgência	
	%	(IC95%)	%	(IC95%)
Sempre-baixa	14,20	(10,20-18,20)	13,32	(9,33-17,30)
Média-baixa	11,72	(9,16-14,27)	9,74	(7,58-11,90)
Média-alta	16,55	(13,10-20,00)	12,49	(9,31-15,66)
Sempre-alta	19,08	(13,43-24,74)	10,31	(6,61-14,00)
valor de p para a tendência*		0,524		0,535

IC95%: Intervalo de confiança de 95%; Pufa: Envolvimento pulpar, ulceração, fístula ou abscesso. *O valor de p para a tendência linear foi derivado de um modelo bruto de regressão logística multinível, considerando as trajetórias de cobertura como um preditor contínuo.

Os modelos multinível para a associação entre a cobertura municipal de saúde bucal e o índice pufa são apresentados na Tabela 3. De acordo com o CPV do modelo nulo, 18,3% da variância do desfecho se deve às diferenças entre os municípios. A inclusão de todas as covariáveis em nível individual no Modelo 1 reduziu a variância em nível municipal em 4,8%, enquanto a adição posterior de preditores em nível municipal, incluindo a cobertura municipal de saúde bucal, explicou 14,7% da variância restante. As crianças de municípios com cobertura média-alta (0,59; intervalo de confiança de 95% - IC95% 0,35-0,98) e média-baixa (0,63; IC95% 0,40-0,99) apresentaram menores chances de ter consequências clínicas de cárie dentária não tratada do que as dos municípios com cobertura sempre-baixa. Contudo, não houve diferença entre as crianças dos municípios com cobertura sempre-alta e sempre-baixa (Tabela 3).

**Tabela 3.** Associação entre a cobertura municipal de saúde bucal e a prevalência de crianças de cinco anos com consequências clínicas de cárie dentária (pufa≥1) (n=7.185 crianças em 351 municípios).

Efeitos fixos	Modelo 0	Modelo 1	Modelo 2
	OR (IC95%)	OR (IC95%)	OR (IC95%)
Cobertura municipal de saúde bucal			
Sempre-baixa			1,00 (Referência)
Média-baixa			0,63 (0,40-0,99)
Média-alta			0,59 (0,35-0,98)
Sempre-alta			0,63 (0,33-1,20)
PIB *per capita* municipal por aumento de R$ 1.000			0,99 (0,98-1,00)
Região geográfica			
Norte			2,22 (1,31-3,78)
Nordeste			1,32 (0,77-2,30)
Sudeste			1,00 (Referência)
Sul			1,56 (0,86-2,83)
Centro-Oeste			1,54 (0,89-2,66)
Tipo de município			
Capital do estado			1,00 (Referência)
Outras cidades			1,27 (0,89-2,66)
Efeitos aleatórios	Estimativas	Estimativas	Estimativas
Variância (EP)	0,74 (0,15)	0,70 (0,16)	0,60 (0,17)
MOR	2,27	2,22	2,09
CPV-nível 2	18,3%	17,6%	15,4%
APV		-4,8%	-14,7%

IC95%: Intervalo de confiança de 95%; PIB: Produto interno bruto em nível municipal; MOR: razão de chance mediana; APV: Alteração proporcional na variância; Pufa: Envolvimento pulpar, ulceração, fístula ou abscesso; OR: *Odds ratio*; EP: Erro padrão; CPV-nível 2: coeficiente de partição de variância em nível municipal.

Um modelo de regressão logística multinível foi ajustado com crianças aninhadas nos municípios. O Modelo 0 foi nulo (sem preditores). O Modelo 1 incluiu todas as covariáveis em nível individual como preditores. O Modelo 2 incluiu todas as covariáveis em nível individual e municipal e a cobertura municipal de saúde bucal como preditores.

Os resultados para a necessidade de tratamento odontológico de urgência são apresentados na Tabela 4. De acordo com o CPV do modelo nulo, 18,0% da variância do desfecho foi devido às diferenças entre os municípios, que diminuiu 4,0% após a inclusão das covariáveis em nível individual no Modelo 1. A posterior inclusão de preditores em nível municipal no modelo, incluindo a cobertura municipal de saúde bucal, representou 6,9% da variância restante (Modelo 2). As crianças dos municípios com cobertura sempre-alta (OR 0,50; IC95% 0,25-0,99) apresentaram menores chances de urgência odontológica do que as dos municípios com cobertura sempre-baixa. Todavia, não houve diferenças entre as crianças dos municípios com cobertura média-alta e média-baixa e as dos municípios com cobertura sempre-baixa (Tabela 4).

**Tabela 4.** Associação entre a cobertura municipal de saúde bucal e a prevalência de crianças de cinco anos com necessidade de tratamento odontológico de urgência (n=7.185 crianças em 351 municípios).

Efeitos fixos	Modelo 0	Modelo 1	Modelo 2
	OR (IC95%)	OR (IC95%)	OR (IC95%)
Cobertura municipal de saúde bucal			
Sempre-baixa			1,00 (Referência)
Média-baixa			0,69 (0,41-1,14)
Média-alta			0,76 (0,43-1,36)
Sempre-alta			0,50 (0,25-0,99)
PIB *per capita* municipal, aumento de R$ 1.000			1,00 (0,99-1,01)
Região geográfica			
Norte			1,63 (0,90-2,96)
Nordeste			1,06 (0,58-1,91)
Sudeste			1,00 (Referência)
Sul			1,29 (0,66-2,54)
Centro-Oeste			1,44 (0,77-2,71)
Tipo de município			
Capital do estado			1,00 (Referência)
Interior			1,05 (0,72-1,53)
Efeitos aleatórios	Estimativas	Estimativas	Estimativas
Variância (EP)	0,72 (0,22)	0,70 (0,25)	0,65 (0,24)
MOR	2,25	2,22	2,15
CPV-nível 2	18,0%	17,4%	16,4%
APV		-4,0%	-6,9%

IC95%: Intervalo de confiança de 95%; PIB: Produto interno bruto em nível municipal; MOR: razão de chance mediana; APV: Alteração proporcional na variância; OR: *Odds ratio*; EP: Erro padrão; CPV-nível 2: coeficiente de partição de variância em nível municipal.

Um modelo de regressão logística multinível foi ajustado com crianças aninhadas nos municípios. O Modelo 0 foi nulo (sem preditores). O Modelo 1 incluiu todas as covariáveis em nível individual como preditores. O Modelo 2 incluiu todas as covariáveis em nível individual e municipal e a cobertura municipal de saúde bucal como preditores.

### DISCUSSÃO

De acordo com os resultados deste estudo, em comparação com crianças residentes em municípios com cobertura de saúde bucal sempre-baixa, aquelas que vivem em municípios com cobertura média-baixa e média-alta foram menos propensas a sofrer consequências clínicas de cárie dentária não tratada, enquanto que aquelas que residem em municípios com cobertura sempre-alta foram menos propensas a precisar de tratamento odontológico de urgência, considerando as covariáveis tanto em nível municipal quanto em nível individual.

A variação geográfica bruta em nível municipal explicou 18,3% da variância individual total no índice pufa e 18,0% com relação à necessidade de tratamento odontológico de urgência. Após considerar a composição sociodemográfica dos municípios (preditores em nível individual), os CPV diminuíram ligeiramente para 17,6 e 17,4%, respectivamente. Considerando que valores de CPV≥5% normalmente são interpretados como refletindo efeitos contextuais moderados^30^, tais resultados sugerem que o contexto municipal desempenha um papel significativo na constituição dos desfechos de saúde bucal entre crianças pequenas. As desigualdades acentuadas em saúde bucal observadas entre os municípios podem refletir variações no investimento a longo prazo em infraestrutura, programas de saúde pública ou acesso à assistência. A inclusão de preditores em nível municipal, incluindo a cobertura municipal de saúde bucal, foi responsável, em certa medida, pela variância total em nível individual no índice pufa (APV=14,7%) e na necessidade de tratamento odontológico de urgência (APV=6,9%), sugerindo que fatores contextuais adicionais, como os determinantes estruturais da saúde, também podem contribuir para essas desigualdades.

Sistemas sociais, políticos e econômicos podem influenciar a saúde bucal das crianças por meio de políticas, alocação de recursos e organização da sociedade. As políticas sociais, incluindo a disponibilidade e a qualidade do ensino público (especialmente durante os anos de maternal e pré-escola), e os sistemas de assistência social (por exemplo, os gastos do governo com saúde e proteção social) criam oportunidades institucionais para a promoção da saúde bucal, prevenção e gestão precoce de doenças dentárias. São, portanto, fundamentais para facilitar o tratamento oportuno e evitar a progressão para emergências dentárias agudas.

De acordo com os nossos resultados, a expansão da cobertura municipal de saúde bucal pode reduzir, mas não elimina completamente as desigualdades geográficas em termos de saúde bucal. A mera disponibilidade de serviços não garante um acesso eficaz, uma vez que as prioridades dos serviços e a distribuição da força de trabalho podem afetar o escopo e a qualidade dos serviços^31^. A variabilidade dos resultados de saúde bucal observada entre os municípios destaca a necessidade de considerar não só a disponibilidade de serviços, mas também a sua eficácia na oferta de cuidados preventivos e restauradores adequados à idade. Outro desafio é o modelo predominante de assistência, no qual os profissionais de saúde bucal atuam principalmente em consultórios odontológicos, com colaboração limitada com o setor da educação para promover a saúde bucal e a prevenção de doenças. Iniciativas governamentais, como o Programa Saúde na Escola, que visa integrar a Atenção Básica aos contextos educativos, têm lutado para alcançar uma implementação consistente e ampla. Abordar essas lacunas é crucial para maximizar o impacto preventivo da cobertura municipal^11^ ^,^ ^12^.

O papel protetor esperado da expansão da cobertura de saúde bucal no SUS tem sido amplamente reconhecido. A ESF e a Política Nacional de Saúde Bucal (Brasil Sorridente) foram criadas para reduzir as desigualdades no acesso à assistência odontológica, e autores de estudos recentes têm documentado a expansão e a consolidação das equipes de saúde bucal na Atenção Básica^32^ ^,^ ^33^. Tais equipes fornecem uma ampla gama de serviços gratuitos, incluindo promoção da saúde, prevenção de doenças bucais, diagnóstico, cuidados restauradores, extrações e atendimento de emergência^13^. Autores de estudos anteriores demonstraram que crianças em áreas atendidas pelas unidades de saúde da ESF têm maior probabilidade de visitar o dentista no início da vida, apresentar melhor qualidade de vida relacionada à saúde bucal e receber mais tratamentos odontológicos (restaurações e extrações) em comparação a outras crianças^34^ ^,^ ^35^. Os achados do presente estudo são significativos porque o período de análise (2019-2023) abrangeu a pandemia de covid-19, que sobrecarregou o sistema de saúde brasileiro de 2020 a 2022. Durante esse período, procedimentos eletivos foram suspensos e as equipes de saúde bucal foram realocadas para atendimentos de urgência. Apesar desses desafios, o tratamento odontológico de urgência continuou a ser prestado ao longo da pandemia.

Tais achados têm algumas implicações em termos de políticas públicas. Os resultados corroboram a noção de que a expansão da cobertura municipal de saúde bucal pode ajudar a reduzir a prevalência de crianças com má saúde bucal. Em um sistema baseado na cobertura universal de saúde, alcançar uma cobertura municipal ampla e equitativa é fundamental para defender os princípios da Atenção Primária à Saúde. O fortalecimento e a manutenção da cobertura de saúde bucal devem, portanto, continuar a ser prioridade para garantir o acesso oportuno à assistência e promover resultados mais equitativos de saúde bucal para crianças pequenas no Brasil.

Este estudo tem algumas limitações. Primeiro, apesar de termos dados longitudinais sobre a cobertura municipal de saúde bucal, a natureza transversal do inquérito SB Brasil nos impede de fazer quaisquer inferências causais sobre o impacto da ESF na saúde bucal infantil. Segundo, para realizar a análise, assumimos que as crianças moravam no mesmo município desde o seu nascimento. A má classificação diferencial da exposição poderia ter ocorrido se os padrões de mobilidade residencial variassem de acordo com desfechos de saúde bucal. Além disso, não podemos excluir a confusão residual, especialmente devido a fatores contextuais não avaliados como a disponibilidade de programas sociais e a fluoretação da água de abastecimento.

No presente estudo, verificamos que, em comparação com as crianças que vivem em municípios com cobertura de saúde bucal consistentemente alta, aquelas que residem em municípios com cobertura média tinham menos probabilidade de sofrer consequências clínicas de cárie dentária não tratada, enquanto as que vivem em municípios com cobertura consistentemente alta foram menos propensas à necessidade de tratamento odontológico de urgência. De acordo com os resultados, crianças que vivem em municípios com cobertura consistentemente baixa estão mais propensas a doenças dentárias graves, porém evitáveis. Em um sistema de saúde fundamentado nos princípios da cobertura universal de saúde, a incapacidade de atender crianças pequenas adequadamente constitui um problema crítico que merece atenção urgente.

## Material Suplementar

Supplementary Material
